# Reply regarding: Change in treatment preferences in pediatric diaphyseal forearm fractures: a Danish nationwide register study of 36,244 fractures between 1997 and 2016 (Hansen et al. Acta Orthop 2023; 94: 32–7)

**DOI:** 10.2340/17453674.2024.40814

**Published:** 2024-06-10

**Authors:** Bjarke Viberg

**Affiliations:** Department of Orthopaedic Surgery and Traumatology, Lillebaelt Hospital Kolding, University Hospital of Southern Denmark; Department of Orthopaedic Surgery and Traumatology, Odense University Hospital, Denmark

*Sir,*—We thank Husum et al. [1] for taking an interest in our paper [2]. As they know and write, register studies have, in similarity with other studies, both strengths and limitations. In the letter to the editor of Acta Orthopaedica they express concerns regarding the validity of the data utilized in our study and consequently, the robustness of our findings. We agree that data validity is an important aspect in register studies as it reflects the quality of the registered data [2]. The validity of data consists of 4 major aspects:

1. coverage of the register;
2. registration completeness of procedures/patients;
3. registration completeness of variables included in the register;
4. accuracy of registered variables.

The data is retrieved from the Danish National Patient Registry (DNPR), which has 100% coverage as it has data from all hospitals in Denmark including the private sector [3]. The completeness in the DNPR is regarded to be 99.7%; hence, the data is more than robust regarding points 1–3. One of the correspondents’ concerns was regarding the accuracy of the registered variables (point 4), which we also described in the discussion as we do not know the accuracy concerning the fracture diagnosis in the pediatric population. In their letter, they refer to more validation studies of orthopedic diagnosis in the DNPR, which in general have high accuracy. We acknowledge that there could be a difference concerning the pediatric population and agree that it is a future research point.

Their concerns are specifically regarding the distribution of fractures as well as the use of K-wires. Our fracture distribution is 15% ulna shaft, 30% radius shaft, and 55% combined. If we compare our results with the data in the Swedish Fracture Register they are fairly close, as they report 10% ulna shaft, 20% radius shaft, and 70% combined [4]. There might be a misclassification of diagnoses, which we know from the studies they refer to and it is usually approximately 10%. In our study, it could be metaphyseal radius fractures being reported/diagnosed as shaft fractures. The surgical codes in all accuracy studies have a very high positive predictive value around 98% and we find the high use of K-wires therefore fairly certain. There are several reasons for including the K-wires as we have stated in the bias section, and one is incorrect diagnosis coding but an incorrect procedure coding is also possible.

Is the data in our study robust? We think it is, as there is the high coverage, high completeness, and high accuracy known from other fracture validation studies. However, we do not know the exact accuracy for the pediatric population. Does it change the main outcome of this study? No, as we assume that a possible accuracy bias would be the same throughout the period analyzed. We find that the conclusion of an increase in invasive treatment due to the use of intramedullary nails to be sound and in correspondence with our clinical observations.

On behalf of the authors

**Bjarke Viberg**

*Department of Orthopaedic Surgery and Traumatology, Lillebaelt Hospital Kolding, University Hospital of Southern Denmark; Department of Orthopaedic Surgery and Traumatology, Odense University Hospital, Denmark*

*Correspondence: bjarke.viberg@rsyd.dk*

## References

[1] Rahbek O, Kold S, Husum H-C. Letter to the editor: Change in treatment preferences in pediatric diaphyseal forearm fractures: a Danish nationwide register study of 36,244 fractures between 1997 and 2016 (Hansen et al. 2023). Acta Orthop 2024: 95: 282. doi: 10.2340/17453674.2024.40813. 36727711 PMC9893835

[2] Hansen R T, Borghegn N W, Gundtoft P H, Nielsen K A, Balslev-Clausen A, Viberg B. Change in treatment preferences in pediatric diaphyseal forearm fractures: a Danish nationwide register study of 36,244 fractures between 1997 and 2016. Acta Orthop 2023; 94: 32-7. doi: 10.2340/17453674.2023.7132. 36727711 PMC9893835

[3] Varnum C, Pedersen A B, Gundtoft P H, Overgaard S. The what, when and how of orthopaedic registers: an introduction into register-based research. EFORT Open Rev 2019; 4(6): 337-43. doi: 10.1302/2058-5241.4.180097. 31210972 PMC6549105

[4] Schmidt M, Schmidt S A, Sandegaard J L, Ehrenstein V, Pedersen L, Sørensen H T. The Danish National Patient Registry: a review of content, data quality, and research potential. Clin Epidemiol 2015; 7: 449-90. doi: 10.2147/CLEP.S91125. 26604824 PMC4655913

[5] Svenska Frakturregistret. Årsrapport 2022. Available from: https://registercentrum.blob.core.windows.net/sfr/r/-rsrapport-SFR-2022-By_ps8PU3.pdf

